# Immigrant Status Predicts Worse Subjective Memory Complaints

**DOI:** 10.1093/geroni/igaa057.1096

**Published:** 2020-12-16

**Authors:** Yujin Franco, Yujin Jeong

**Affiliations:** University of Southern California, Los Angeles, California, United States

## Abstract

The “Immigrant Health Paradox” suggests that immigrants experience better health and lower mortality than the U.S.-born population despite their lower average socioeconomic status. However, it is unclear whether this health advantage extends to all areas of cognitive functioning. This study investigates cognitive functioning and Subjective Memory Complaints (SMCs) as a function of immigrant status and identifies predictors of cognitive decline as well as SMCs among the immigrant population. Data were drawn from the 2010 wave of the Health and Retirement Study. The sample consisted of 9,812 older adults aged 65 and older (8,873 U.S.-born and 939 foreign-born). Logistic regression was used to examine whether immigrant status was associated with cognitive functioning and SMCs, controlling for socio-demographic (age, gender, education, and marital status), health conditions (diabetes, depression, hypertension, and stroke), and functional limitations. Being foreign-born was not a significant predictor of dementia (OR:1.18, 95% CI: 0.83-1.67). However, immigrants were 41% more likely to report SMCs compared to U.S.-born respondents (OR:1.41, 95% CI: 1.17-1.69). Among the immigrant population, immigrants with less than a high school education showed four- and two-times higher odds of having dementia and reporting SMCs than those with more than high school. It is necessary to provide dementia education and screening to immigrants, especially those with low education, as this may contribute to reducing disparities in cognitive functioning within the older population.

